# Peripheral BDNF Levels in Individuals at Ultra-High Risk for Psychosis: A Systematic Review

**DOI:** 10.3390/brainsci15090928

**Published:** 2025-08-27

**Authors:** Omar Contreras, Carla Rivera, Carolina Villaseca, Francisco Mas, Benjamín Cartes, Rolando Castillo-Passi, Rodrigo R. Nieto

**Affiliations:** 1 Escuela de Medicina, Facultad de Medicina, Universidad de Chile, Santiago 8380453, Chile; 2 Clínica Psiquiátrica Universitaria, Hospital Clínico de la Universidad de Chile, Santiago 8380453, Chile; 3 Departamento de Psiquiatría, Psicología y Neurología, Clínica Alemana Universidad del Desarrollo, Santiago 7650567, Chile; 4 Laboratorio de Psiquiatría Traslacional Psiquislab, Departamento de Psiquiatría Norte, Hospital Clínico Universidad de Chile, Santiago 8380453, Chile; 5 Departamento de Psiquiatría y Salud Mental, Facultad de Medicina, Universidad de Chile, Santiago 8380453, Chile; 6 Departamento de Neurociencias, Facultad de Medicina, Universidad de Chile, Santiago 8380453, Chile; 7 Departamento de Psiquiatría Adultos, Clínica Las Condes, Santiago 7591047, Chile

**Keywords:** ultra-high risk, brain-derived neurotrophic factor, peripheral biomarkers, first-episode psychosis, schizophrenia

## Abstract

**Background/Objectives**: Brain-derived neurotrophic factor (BDNF) is a neurotrophin critical for neurogenesis and synaptic plasticity, and alterations in its peripheral levels have been associated with schizophrenia and other psychotic disorders. However, findings on peripheral BDNF levels in individuals at ultra-high risk (UHR) for psychosis have been inconsistent. This review synthesizes current evidence comparing peripheral BDNF levels in UHR populations with those in healthy controls (HCs), first-episode psychosis (FEP), and chronic schizophrenia (CS), focusing on BDNF’s potential relevance as a biomarker of psychosis risk and subsequent clinical course. **Methods**: A systematic search of PubMed, Scopus, and Web of Science identified studies reporting baseline peripheral BDNF levels in UHR individuals compared with HC, FEP, or CS. Of 755 records retrieved, 608 unique titles/abstracts were screened, 49 full texts reviewed, and 8 studies included. Two reviewers independently screened, extracted data, and assessed risk of bias. Given marked clinical and methodological variability, results were synthesized narratively. **Results**: Eight studies met eligibility criteria and were synthesized across three analytical categories: (1) UHR vs. HC; (2) UHR vs. FEP or CS; and (3) longitudinal outcomes. Findings were inconsistent; some studies reported lower BDNF in UHR relative to comparison groups, whereas others found no differences or higher levels, often influenced by clinical or methodological factors. Longitudinal analyses did not reveal consistent prognostic value, and heterogeneity precluded meta-analysis. **Conclusions**: Findings across studies were inconsistent and limited by small samples, as well as by methodological heterogeneity. While current evidence does not support its prognostic use, peripheral BDNF may still hold potential as part of a biomarker framework if evaluated in larger, standardized, and rigorously controlled studies.

## 1. Introduction

Schizophrenia and related psychotic disorders are chronic, disabling conditions associated with progressive cognitive decline, poor functional outcome, and significant global disease burden [1,2,3,4,5,6,7]. Early detection and intervention have been associated with improved outcomes and reduced morbidity, particularly when initiated during the prodromal phase [8,9,10,11,12]. Over the past three decades, the ultra-high risk (UHR) paradigm has emerged as a prominent clinical framework for identifying individuals at elevated risk for psychosis. It has also been used to support investigations into early pathophysiological mechanisms and the development of preventive interventions [8,9,10,11,12].

In parallel with clinical staging approaches such as the UHR paradigm, peripheral biomarkers have emerged as a focus of investigation for their potential role in early detection and disease monitoring [13,14,15]. One biomarker that has received considerable attention is brain-derived neurotrophic factor (BDNF), due to its well-documented involvement in neurodevelopment, synaptic plasticity, and cognitive function [16,17]—processes known to be disrupted in psychosis [18,19,20]. Consistent evidence indicates that individuals with first-episode psychosis (FEP) and chronic schizophrenia (CS) exhibit reduced peripheral BDNF levels, suggesting a link between neurotrophic deficits and the pathophysiology of psychotic disorders [20,21,22,23].

Recent literature further highlights the cognitive implications of BDNF dysregulation. Peripheral BDNF level alterations have been related to impaired cognition in schizophrenia and psychosis, particularly in memory, attention, and executive functioning domains [21], supporting the potential utility of BDNF not only as a pathophysiological marker but also as a biomarker of cognitive outcomes in psychotic disorders [21]. Additionally, emerging evidence suggests that peripheral BDNF levels may vary across different stages of psychotic illness and treatment, potentially reflecting underlying neurobiological processes associated with illness progression [20,21,22,23,24].

In the context of these findings, scientific interest in whether BDNF alterations are already present in UHR individuals has emerged. Nevertheless, studies examining peripheral BDNF levels in UHR populations have produced conflicting results. Some studies report decreased concentrations compared to healthy controls (HCs) [25,26], consistent with models of early neurobiological vulnerability [16,18]. Others report no significant differences [27,28] or even higher BDNF levels [29]. Such discrepancies may in part reflect methodological and clinical sources of heterogeneity, as previously reported in research on peripheral BDNF levels in schizophrenia. These include variation in diagnostic definitions, biological sample types, assay platforms, and exposure to psychotropic medication [15,22,23,24,25,26,29].

This review aims to systematically examine and narratively synthesize the current evidence on peripheral BDNF levels in individuals at ultra-high risk for psychosis. Specifically, it compares peripheral BDNF levels among ultra-high-risk individuals, healthy controls, and patients with first-episode psychosis or chronic schizophrenia, to assess whether alterations are already detectable during the early or prodromal phase. By doing so, the review evaluates the potential of BDNF as a biomarker of risk for psychosis and its clinical course.

## 2. Materials and Methods

*Protocol and Registration*—This systematic review was conducted in accordance with the Preferred Reporting Items for Systematic Reviews and Meta-Analyses (PRISMA 2020) guidelines [30]. The review protocol was registered in the International Prospective Register of Systematic Reviews PROSPERO; registration ID: CRD420251072963.

*Eligibility Criteria*—Eligible studies had to meet the following inclusion criteria. First, they reported original quantitative data on BDNF in human subjects, with no restrictions on the biological matrix. Second, they compared BDNF levels at baseline between individuals classified as UHR—including those meeting criteria for at-risk mental state (ARMS), attenuated positive symptoms (APS), brief limited intermittent psychotic symptoms (BLIPS), genetic risk and deterioration (GRD) or basic symptom (BS) criteria—and at least one of the following comparison groups: HC, individuals with a FEP, or individuals with CS. We did not consider studies measuring immature forms or precursors of BDNF (e.g., proBDNF, BDNF mRNA) as meeting the criterion for measurement of BDNF, because these are biologically distinct from the mature protein and have been associated with different circulating levels and distinct biological effects [31,32].

*Information Sources and Search Strategy*—A systematic literature search was conducted in PubMed, Web of Science, and Scopus to identify studies on BDNF levels in individuals at UHR. No language restrictions were applied. The search strategy combined terms for BDNF with terms for the UHR construct and psychosis. A prototype search string was (BDNF OR “brain-derived neurotrophic factor”) AND (“ultra high risk” OR “ultra-high risk” OR UHR OR “clinical high risk” OR “clinical high-risk” OR CHR OR “at risk mental state” OR “at-risk mental state” OR “attenuated psychosis” OR “attenuated psychosis syndrome” OR APS OR “basic symptoms” OR BLIPS OR “sub syndromal” OR subsyndromal OR prodrom* OR “prodromal psychosis”) AND (psychosis OR schizophrenia). Database-specific syntax and field tags were adjusted as required. The search was performed iteratively and finalized on 14 August 2025 to ensure completeness and accuracy. Additionally, the reference lists of all included articles were screened to identify further eligible studies.

*Selection Process*—Two reviewers independently screened titles and abstracts and assessed full-text articles for eligibility; disagreements were resolved through consultation with a third reviewer. No automation tools were used during screening. At the abstract screening stage, inter-rater reliability between the two reviewers was moderate (κ = 0.54), with very high raw agreement (574/608; 94%). The discrepancy between κ and raw agreement was likely due to the strong imbalance between the large number of exclusions and the small number of inclusions [33].

*Data Extraction*—Data extraction was conducted independently by two reviewers using a standardized table; as all items were numeric or explicitly reported, no discrepancies arose. No automation tools were used. From each eligible study, the data extracted included title, authors, and year of publication; sample sizes, UHR selection criteria for each diagnostic group (UHR, FEP, CS, and HC); and average age of participants. Where reported, body mass index (BMI), use of antipsychotics, use of antidepressants, and use of tobacco or nicotine were also recorded. For each diagnostic group, mean BDNF levels and *p*-values for group comparisons were collected, along with whether a follow-up was conducted. The type of biological sample used for BDNF measurement was also recorded when applicable. The primary outcome for this review was the difference in BDNF levels between UHR and the comparison groups, based on these reported means.

*Risk of Bias Assessment*—Risk of bias in included studies was assessed using the Joanna Briggs Institute (JBI) Critical Appraisal Checklist for Analytical Cross-Sectional Studies [34], which is appropriate for observational, cross-sectional designs. Two reviewers independently assessed all studies in duplicate. Disagreements were resolved through discussion, with a third reviewer consulted if consensus could not be reached. We did not formally assess reporting bias (e.g., publication bias), consistent with the descriptive, narrative approach of this review.

*Data Synthesis*—Studies were eligible for synthesis if they reported mean BDNF levels for UHR individuals and at least one comparison group (HC, FEP, or CS). Eligible studies were grouped according to the diagnostic categories compared within each study. No additional data preparation or conversions were performed; data were extracted exactly as reported by the original authors. Results of individual studies were tabulated in structured summary tables showing reported mean BDNF levels and available statistical comparisons. A qualitative, narrative synthesis was conducted to summarize findings across studies. A quantitative synthesis (meta-analysis) was not conducted due to substantial variability in both study methods and participant characteristics. Sources of variability included differences in diagnostic instruments used to define UHR status (e.g., CAARMS vs. SIPS), measurement matrices (serum vs. plasma), sample sizes and statistical power, clinical characteristics of participants (e.g., antidepressant or antipsychotic use), and availability and duration of follow-up data. No formal statistical assessment of heterogeneity was performed because no meta-analysis was conducted. No subgroup analyses, meta-regression, or sensitivity analyses were undertaken.

## 3. Results

### 3.1. Study Selection

**Figure 1:**
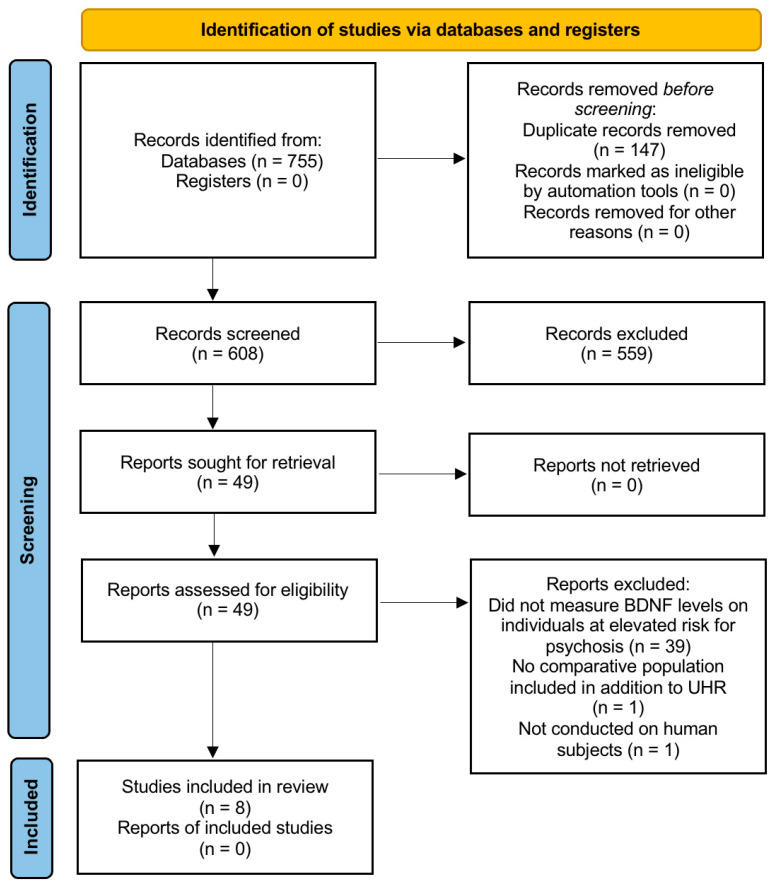
PRISMA (Preferred Reporting Items for Systematic Reviews and Meta-Analyses) flow diagram that illustrates the study selection process for this systematic review. A total of 755 records were identified from database searches, with 147 duplicates and no ineligible records removed before screening. After screening, 559 records were excluded, leaving 49 reports for full-text assessment. No reports were unavailable for retrieval. Of the 49 assessed works, 41 were excluded for reasons including not measuring BDNF in the target population (*n* = 39), not measuring BDNF on healthy controls or subjects with at least one episode of psychosis (*n* = 1), and not involving human subjects (*n* = 1). Ultimately, 8 studies met inclusion criteria and were incorporated into the review.

The database search across PubMed, Scopus, and Web of Science identified 755 records. After removing 147 duplicates, 608 unique records remained. Title and abstract screening excluded 559 records that did not involve peripheral BDNF measurement in individuals at ultra-high risk for psychosis. Forty-nine reports were assessed in full. Of these, forty-one were excluded. Thirty-seven did not report peripheral BDNF measurement in UHR samples; two lacked a comparator group; one examined gene expression rather than protein concentration; and one involved non-human subjects. Eight studies met all eligibility criteria and were included in the final synthesis. Figure 1 presents the PRISMA 2020 flow diagram summarizing identification, screening, eligibility, and inclusion.

### 3.2. Study Characteristics

All eight included studies employed observational designs and examined peripheral BDNF levels in individuals meeting criteria for UHR. UHR status was defined using the Structured Interview for Psychosis-Risk Syndromes (SIPS) in four studies [27,28,35,36], the Comprehensive Assessment of At-Risk Mental States (CAARMS) in three [25,29,37], and the Basel Screening Instrument for Psychosis (BSIP) in one [26].

Comparison groups varied across studies: seven included healthy controls [25,27,28,29,35,36,37], five included first-episode psychosis patients [26,28,35,36,37], and two included chronic schizophrenia patients [26,28]. Counotte et al. [37] additionally included a group of unaffected siblings of psychosis patients, which was not considered for comparison in the present review.

Sample sizes ranged from 33 participants in Heitz et al. [25] to 211 in Yee et al. [28], with considerable variation in the distribution of diagnostic groups. In some samples, UHR participants represented the largest group [26,27]; in others, distributions were balanced [29,36]; and in several, UHR individuals accounted for a smaller proportion [25,28,35,37].

Mean participant age ranged from 20 years in He et al. [36] to 32 years in Cecerska-Heryć et al. [28], and overall there was a predominance of male participants. Recruitment, when considered across all eight studies, spanned Europe [25,26,28,37], North America [35], South America [27], and Asia [29,36]. Ethnicity of participants was specified in two studies [29,35]. Years of education were reported in four studies [25,26,27,37], with significant group differences observed only in Counotte et al. [37], where both UHR and psychosis patients showed lower educational attainment than healthy controls.

Various known modulators of BDNF expression were addressed inconsistently as potential confounders. BMI was documented for only three of the eight samples [28,29,37]. Smoking status, in contrast, was frequently recorded, with six studies providing information on participants’ tobacco or nicotine consumption [25,26,27,28,29,37]. Psychotropic medication use was addressed unevenly. One study, Yee et al., excluded participants receiving antipsychotics and was also the only one to provide detailed information on antidepressant use [29]. Four studies reported antipsychotic use but did not mention other psychotropic medications [25,26,28,35]. One described psychotropic use without specifying the class [37], and two provided no information on medication use among their participants [27,36]. None included data on participants’ levels of physical activity [25,26,27,28,29,35,36,37].

BDNF was quantified using samples from serum and plasma. Three studies used serum for their analyses [28,29,37], four used plasma [25,27,35,36], and one, Heitz et al. [26], used both. Assay techniques varied. Six studies used ELISA [25,26,27,28,29,35], one used a Luminex multiplex assay [37], and one did not specify the method [36].

Outcomes were presented either as mean BDNF concentrations or as statistical comparisons, with varying levels of detail. Table 1 presents a structured overview of these characteristics.

### 3.3. Risk of Bias

Risk of bias was assessed for all eight included studies using the JBI Critical Appraisal Checklist for Analytical Cross Sectional Studies [34]. The assessment was conducted to inform the interpretation of findings and evaluate methodological quality. All studies met basic quality criteria and were retained for synthesis.

### 3.4. Results of Individual Studies

All included studies reported peripheral BDNF concentrations in serum or plasma, either as a primary outcome or as part of a broader set of biomarker analyses. The individual results—comprising group-level BDNF values, reported statistical comparisons, and key methodological and clinical variables—are summarized in Table 1. These findings are narratively synthesized in Section 3.4.

### 3.5. Synthesis of Results

To structure the synthesis and enable clinically meaningful comparisons, studies were grouped based on the diagnostic samples they included and the comparisons they allowed. Since all studies involved individuals at UHR, we categorized them according to whether they also included HC, FEP, and/or CS participants. This approach enabled two main types of comparison: (1) UHR versus HC, and (2) UHR versus individuals with established psychotic disorders (FEP/CS). We also established a third analytical category, comprising studies that followed UHR participants longitudinally to examine whether baseline BDNF levels were associated with subsequent clinical outcomes. This grouping strategy reflects the structure of the available data and allows examination of the hypothesis that peripheral BDNF levels may vary in parallel with the clinical progression of psychosis [20,22,38,39].

#### 3.5.1. Comparison Between UHR and HC

Seven studies [25,27,28,29,35,36,37] compared peripheral BDNF levels between UHR and HC participants. Findings were inconsistent. Two studies [25,28] reported lower BDNF levels in UHR participants, though only one reached statistical significance [25]; three studies found no difference [27,36,37]; and two reported higher levels in UHR samples [29,35].

Sanada et al. [25] (*n* = 13 UHR, 30 HC) and Cecerska-Heryć et al. [28] (*n* = 13 UHR, 34 HC) both reported lower peripheral BDNF levels in UHR, though only Sanada et al. found a statistically significant difference (3.86 vs. 12.92 ng/mL, *p* = 0.001). Cecerska-Heryć et al. observed a similar directional trend (21 pg/mL in UHR vs. 35 pg/mL in HC), but the difference did not reach statistical significance (*p*-value not reported) [28]. Cecerska-Heryć et al. [28] analyzed serum samples, whereas Sanada et al. [25] analyzed plasma, with both studies using ELISA for quantification.

These results are consistent with the hypothesis that peripheral BDNF levels decrease along the clinical course of psychotic illness. Their interpretation, however, is limited primarily by the very small sample sizes, which reduce statistical power and make the findings less robust. In addition, the high prevalence of psychotropic medication use represents a potential source of confounding that further complicates interpretation. Antipsychotic use was reported in eight of thirteen (61.5%) UHR participants in Sanada et al. [25] and ten of thirteen (76.9%) in Cecerska-Heryć et al. [28]. Antidepressant use was not documented in either study, despite its well-documented impact on BDNF expression [40,41,42].

Yee et al. [29] (*n* = 105 UHR, 106 HC) and Kelsven et al. [35] (*n* = 11 UHR, 10 HC) both found higher BDNF levels in UHR compared to HC. Yee et al. reported serum concentrations of 3.7 vs. 3.3 ng/dL (*p* = 0.018) [29], whereas Kelsven et al. observed plasma values of 18.08 vs. 3.87 ng/mL (*p* < 0.001) [35]. Both used ELISA for quantification.

The study by Yee et al. [29] is noteworthy for several reasons. First, it featured the largest sample among the included studies, with nearly equal numbers of UHR and HC participants. Second, it excluded individuals receiving antipsychotics, thereby eliminating a major source of confounding. Most notably, it was the only study to provide detailed information on antidepressant use. On this basis, the authors conducted a stratified analysis showing that the difference in BDNF levels between UHR and HC was primarily driven by the 59 UHR participants not on antidepressants (56.2%). In contrast, the 46 UHR participants receiving antidepressant treatment (43.8%) showed no significant difference from HC (*p* = 0.821) [29].

The study by Kelsven et al. [35] was much smaller but also featured a balanced number of UHR and HC participants. Use of antidepressants was not reported. Although some participants in the overall sample were taking antipsychotics, none of the UHR or HC participants were. Thus, along with Yee et al. [29], it was one of only two studies that compared UHR and HC groups free of antipsychotic exposure.

These findings appear to be in conflict with the notion of a linear decline in BDNF across the course of psychotic illness, and instead align with the view that early or high-risk stages may involve a compensatory response reflected in rising BDNF levels [16,32].

Finally, Loch et al. [27] (*n* = 81 UHR, 55 HC), Counotte et al. [37] (*n* = 11 UHR, 39 HC), and He et al. [36] (*n* = 13 UHR, 30 HC) reported no significant differences in BDNF levels between UHR and HC groups.

Despite their similar findings, the three studies differed in various aspects. While Counotte et al. and He et al. had relatively similar small sample sizes [36,37], Loch et al. included a much larger cohort [27]. They also differed in their biological matrices and quantification techniques: Loch et al. and He et al. obtained samples from plasma [27,36], whereas Counotte et al. used serum [37]; Loch et al. and Counotte et al. employed ELISA for quantification [27,37], while He et al. did not specify any method [36]. Importantly, Counotte et al. did not treat BDNF levels by themselves as a primary outcome.

The interpretability of these findings is constrained mainly by methodological limitations. On the one hand, two studies (Counotte et al. [37] and He et al. [36]) relied on very small samples. On the other hand, there was an incomplete reporting of potential confounders, which makes the results more difficult to interpret. This issue is particularly relevant for Loch et al. [27] and He et al. [36], which provided the least information in this respect. Of the four potential confounding factors on BDNF expression we extracted data for—BMI, antipsychotic use, antidepressant use, and tobacco/nicotine use—Loch et al. [27] reported only on tobacco/nicotine use, while He et al. [36] reported none.

#### 3.5.2. Comparison Between UHR and FEP/CS

Five studies [26,28,35,36,37] compared peripheral BDNF levels between UHR individuals and patients with psychotic disorders (FEP or CS). Results were heterogeneous. Two studies [26,36] reported lower BDNF levels in UHR compared to psychosis groups; one study [35] observed higher levels in UHR; and two found no significant differences [28,37].

Two studies found significantly lower peripheral BDNF levels in UHR compared with patients with established psychosis. Heitz et al. [26] (*n* = 16 UHR, 6 FEP, 11 CS) reported lower serum concentrations in UHR participants compared with both FEP (19.11 vs. 24.48 ng/mL) and CS (19.22 vs. 28.08 ng/mL). After adjustment for age, nicotine use, and antipsychotic dose expressed in chlorpromazine equivalents, these differences remained statistically significant in serum (*p* = 0.015) and showed a suggestive trend in plasma (*p* = 0.09) [26]. Similarly, He et al. [36] (*n* = 30 UHR, 30 FEP) observed lower plasma BDNF in UHR than in FEP (11.43 vs. 12.59 ng/mL, *p* < 0.001).

These findings contrast with the hypothesis of a strictly linear decline in BDNF levels across illness stages, in which UHR would show higher concentrations than FEP or CS.

The interpretability of these results is limited by methodological issues, however: the small sample sizes in Heitz et al. [26] and the lack of reporting on potential confounders in He et al. [36]. Nonetheless, Heitz et al. has notable strengths, including the use of both plasma and serum measures, as well as adjustment for relevant covariates [26].

One study, Kelsven et al. [35] (*n* = 11 UHR, 50 FEP), reported higher plasma BDNF concentrations in UHR participants compared with those with FEP (18.08 vs. 5.01 ng/mL, *p* < 0.001), a finding aligned with the hypothesis of a linear decline in BDNF levels across illness stages. A notable feature of this study is that only 10 participants across the UHR and FEP groups were receiving antipsychotics, all from the FEP group. This corresponds to 14.1% of the total sample when including healthy controls (*n* = 71), which is considerably lower than in most other studies in this review. However, other potential confounders such as BMI, nicotine/tobacco use, and antidepressant treatment were not addressed [35].

Two studies found no significant differences in peripheral BDNF levels between UHR and patients with psychotic illness. Cecerska-Heryć et al. [28] (*n* = 13 UHR, 31 FEP, 72 CS) found no statistically significant difference between UHR and both FEP and CS groups. However, while statistical comparisons were reported by the authors, exact serum BDNF values were not provided numerically and were only shown graphically, limiting the interpretability of the results.

Counotte et al. [37] (*n* = 11 UHR, 38 FEP) likewise reported no significant differences in serum BDNF concentrations between groups. As previously noted, the interpretability of these findings is limited by the fact that BDNF was not a primary outcome in this study [37].

#### 3.5.3. Longitudinal Associations Between BDNF Levels and Clinical Outcomes in UHR Individuals

Three included studies—Loch et al. [27], Yee et al. [29], and Sanada et al. [25]—followed UHR participants longitudinally to examine whether baseline peripheral BDNF levels were linked to later clinical trajectories. While differing in duration, outcome classification, and measurement methodology, all sought to explore potential relationships between initial BDNF levels and diagnostic progression or symptom evolution over time.

Loch et al. [27] conducted a 30-month follow-up of 81 UHR participants, identified using the Structured Interview for Prodromal Syndromes. Over the follow-up, fifteen individuals (18.5%) transitioned to a psychiatric diagnosis; eleven (13.6%) were diagnosed with affective or anxiety disorders and four (4.9%) developed psychosis. Baseline plasma BDNF levels were slightly lower in individuals who converted (511.42 ng/mL) compared to non-converters (535.8 ng/mL), although this difference was not statistically significant (*p* = 0.596).

Yee et al. [29] followed 71 of 105 UHR participants over 24 months as part of the LYRIKS study. During follow-up, 35 participants (49.3%) achieved symptomatic remission, 26 (36.6%) remained symptomatic, and 10 (14.1%) transitioned to psychosis. Baseline BDNF levels showed no significant association with any clinical outcome, including transition to psychosis or remission.

Sanada et al. [25] conducted a six-month follow-up study involving 13 UHR participants and 30 healthy controls. Plasma BDNF was assessed at baseline and at the end of the follow-up period. At baseline, BDNF levels were significantly lower in the UHR group compared to healthy controls (3.86 vs. 12.92 ng/mL, *p* < 0.001). After six months, mean BDNF levels in the UHR group increased to 6.6 ng/mL; however, this change was not statistically significant. No participants transitioned to psychosis during the study period, and no clear clinical correlates of BDNF change were identified. The small sample size and short follow-up duration limit the interpretability of these longitudinal trends.

## 4. Discussion

### 4.1. General Interpretation of Findings

Although meta-analytic evidence consistently shows that peripheral BDNF levels are lower in schizophrenia compared to healthy controls [20,24], variability at the level of individual studies persists [22,39]. Our review sought to examine whether these inconsistencies extended to the earliest clinical stages of psychosis by focusing on UHR populations and comparing them with healthy controls and patients with first-episode psychosis or chronic schizophrenia.

Review of the included studies revealed no consistent pattern of peripheral BDNF levels across the comparisons involving UHR samples.

These findings reflect the persistent variability in individual study results, and contrast with the concept of peripheral BDNF as a clinically informative biomarker for disease activity in schizophrenia spectrum disorders, expected to decline as illness burden increases. This implied gradient—lower BDNF with increasing illness severity—was not observed in our comparisons of UHR individuals with healthy controls and established psychotic disorders. Across the twelve pairwise comparisons derived from the eight included studies, five showed no significant differences between groups [27,28,36,37], two supported the hypothesis of decreasing BDNF with greater illness severity [25,35], and four contradicted it by reporting findings inconsistent with a linear decline [26,29,35,36].

In line with the absence of a consistent pattern across the cross-sectional observations, the three studies that conducted longitudinal assessments found no prognostic value for baseline peripheral BDNF in UHR populations [25,27,29]. These studies aimed to determine whether initial BDNF levels could predict clinically meaningful outcomes, including transition to psychosis, diagnostic progression, or symptomatic remission [25,27,29], but the observed associations were weak and non-significant. This absence of clear prognostic utility is consistent with broader literature that has examined peripheral BDNF as a potential clinical biomarker, yet has not produced conclusive evidence for its role in predicting psychosis [21,22,38,39].

These findings should be interpreted in the context of the marked heterogeneity of the included studies, which involved differences in both methodology and sample composition. In the following sub-sections, potential sources of this heterogeneity are examined in detail, in an attempt to clarify how they may have shaped the observed results.

### 4.2. Diagnostic Criteria and Clinical Subgroups

Three structured interview tools were used across the included studies: the Comprehensive Assessment of At-Risk Mental States (CAARMS), the Structured Interview for Prodromal Syndromes (SIPS), and the Basel Screening Instrument for Psychosis (BSIP). All are standard instruments within the current UHR paradigm [12]. Each test assesses for genetic risk and deterioration syndrome, brief limited intermittent psychotic symptoms, and attenuated psychotic symptoms. Inclusion as UHR requires the presence of one or more of these criteria, regardless of which one or which combination of them [12]. This allows for clinical variability within UHR samples, which may also extend to phenotypical differences.

Beyond this heterogeneity derived from the UHR criteria themselves, the choice of diagnostic interview tool may also influence sample composition. The way the three instruments used across the included studies assess the presence of GRD, BLIPS, and APS is conceptually similar but not identical [12]. For example, one comprehensive study comparing CAARMS with SIPS, while finding substantial agreement between the tests, highlighted the BLIPS subgroup as a notable exception, mostly due to differences in its operationalization [43].

Taken together, the inclusion of individuals who present GRD, BLIPS, APS, or any combination thereof, and the differing assessment of these domains across interview tools, introduce considerable variability into UHR samples. In practice, this may be reflected in differing proportions of GRD, BLIPS, and APS subgroups across studies, an effect that could become especially pronounced in smaller samples. A key implication for biomarker research is that it remains unknown whether UHR subgroups defined by GRD, BLIPS, APS, or BS differ in neurobiological features and outcomes [12]. In particular, it has been suggested that UHR individuals presenting with cognitive decline and negative symptoms may differ in their biological and prognostic profiles from those without such features [44]. If these or other subgroup differences exist, they could plausibly extend to biomarkers like BDNF, meaning that variation in subgroup composition across studies may have contributed to the inconsistent findings observed in this review.

Overall, schizophrenia may represent a more homogeneous diagnostic entity than UHR, which could account for the consistent BDNF level findings in schizophrenia but the more variable results in UHR.

### 4.3. Genetic Factors and Variability in BDNF Expression

Another possible source of heterogeneity across studies comes from the underlying genetic profiles of the samples, which remain insufficiently characterized and thus raise uncertainty about how genetic differences may have contributed to variability in BDNF findings.

Genotype is a well-documented factor in BDNF activity and expression [31,45,46]. In particular, the functional single nucleotide polymorphism rs6265 (C to T, Val66Met) located in the prodomain region of the BDNF gene has been linked to disruption of intracellular trafficking of mRNA BDNF and reduced neurotrophin secretion [47]. More importantly for the present review, individuals with the CC genotype for rs6265 (Val/Val) have been shown to exhibit significantly higher BDNF protein levels than those with the TT genotype (Met/Met) [47,48].

The global prevalence of the rs6265T allele, associated with disruption of the BDNF pathway, is approximately 19% [47]. Consequently, the likelihood of study samples—particularly larger ones—containing individuals with this allele is considerable. Moreover, its distribution varies markedly across ethnicities, with reported prevalences ranging from 1% in African populations to 45% in Asian populations [47,49]. Such population-level differences in allele frequency may therefore represent a further source of variability in BDNF findings across studies. Notably, the studies included in this review recruited participants from China, Spain, Switzerland, Brazil, Poland, the United States, Mexico, Singapore, and the Netherlands [25,26,27,28,29,35,36,37]. Only two of them reported participant ethnicity in their demographic characteristics [29,35].

None of the included studies employed any form of genotyping, leaving open the possibility that unmeasured genetic variation contributed to the inconsistent findings.

### 4.4. Pharmacological Influences on BDNF Expression

There is considerable evidence on the influence of psychotropic medication on BDNF levels [20,24,40,41,42]. In particular, the effects of both antipsychotics and antidepressants (SSRIs and SNRIs) have been studied extensively across different clinical populations, including schizophrenia [20,24] and major depressive disorder [40,41,42]. Meta-analyses examining the effect of antipsychotics on BDNF levels in schizophrenia have yielded somewhat conflicting results [20,24], whereas studies on the effect of antidepressant treatment in major depressive disorder consistently show that SSRIs and SNRIs increase peripheral BDNF levels [40,41,42,50,51,52].

The influence of psychotropic medication on BDNF expression was addressed variably across studies. Of the eight included, four reported antipsychotic use without mention of antidepressants [25,26,28,35]. One study excluded individuals receiving antipsychotics and reported antidepressant use in its sample [29]. Another noted the use of “psychotropic medication” without further specification [37], and two did not report psychotropic medication use among participants [36,44].

The limited attention to antidepressant exposure—acknowledged in only one of eight studies—represents a likely source of heterogeneity. This omission is particularly relevant given that many individuals meeting criteria for UHR also present with comorbid anxiety or depression, conditions commonly treated with antidepressants [12]. Yee et al. [29], the only study that reported antidepressant use, illustrates both the high frequency of antidepressant exposure in UHR individuals and the extent to which this factor can shape BDNF findings. Nearly half of their UHR participants were receiving antidepressants [29]. Through stratified analyses, they showed that the apparent elevation of BDNF they found in UHR relative to HC was mainly driven by those not on antidepressants, while participants receiving antidepressants showed no significant difference from controls [29].

This specific finding appears to contradict the well-established direction of antidepressant effects on BDNF levels. However, it derives from a post hoc sub-analysis rather than a primary outcome and should be interpreted accordingly. Its importance lies in showing that antidepressant exposure clearly influenced the results of the study. This raises reasonable speculation about the extent to which antidepressant exposure may also have shaped findings in the other included studies.

### 4.5. Lifestyle and Metabolic Influences on BDNF Expression

Lifestyle and metabolic factors—including body weight, nicotine use, physical activity, and sleep—have all been shown to influence peripheral BDNF expression [53,54,55,56,57]. While many of the studies included in this review acknowledged one or more of these variables, fully addressing them remains challenging. Some, such as BMI, can be measured relatively straightforwardly, whereas others—like stress, physical activity, or sleep—are harder to capture consistently in clinical samples. This increases the likelihood of residual confounding and the variability in how these influences are addressed, which in turn contributes to the heterogeneity across studies.

### 4.6. Potential Pathophysiological Mechanisms Underlying BDNF Variability in UHR

In addition to methodological and clinical variability, biological mechanisms may also underlie the inconsistent findings regarding peripheral BDNF levels in UHR populations. Lower levels of BDNF in some UHR individuals may reflect the effects of chronic stress and hypothalamic–pituitary–adrenal (HPA) axis dysregulation, processes that have been associated with reduced BDNF expression through epigenetic modifications such as promoter hypermethylation [32]. Inflammatory activity and oxidative stress—both of which have been also described in UHR—can further suppress BDNF synthesis or interfere with the cleavage of proBDNF into its mature form, thereby reducing its peripheral availability [28,47]. Conversely, higher peripheral BDNF levels observed in other UHR samples may represent an early compensatory response aimed at preserving synaptic plasticity in the context of emerging neurobiological dysfunction [16,32].

### 4.7. Limitations of the Evidence

A key limitation of the available evidence is the predominantly small and uneven sample sizes across the eight included studies, which ranged from 11 to 105 UHR participants. Such restricted cohorts limit statistical power, increase the risk of unstable estimates, and constrain the generalizability of the findings. This limitation was most evident in the longitudinal analyses, where clinical events remained sparse despite follow-up durations of 6 to 30 months: Loch et al. [27] followed 81 UHR individuals for 30 months documenting 15 diagnostic transitions, with psychosis occurring in just four cases; Yee et al. [29] followed 71 of 105 baseline participants for 24 months and recorded 10 transitions to psychosis; and Sanada et al. [25], with only 6 months of follow-up, reported none. Together, the small cohorts and low number of events limit the strength of the evidence regarding the prognostic value of peripheral BDNF at the UHR stage.

Differences in methodology and clinical characteristics of samples also limit the comparability of results across studies. BDNF was quantified using different biological matrices—serum [28,29,37], plasma [25,27,36,52], or both [26]—and ELISA kits varied in manufacturer, calibration standards, and sensitivity, with pre-analytical conditions inconsistently reported. Clinical variability was also present: UHR status was defined using CAARMS [25,29,37], SIPS [27,28,35,36], or BSIP [26], and the set of comparator groups differed across studies, with some including FEP or CS in addition to healthy controls. These differences reduce cross-study comparability and likely contributed to the inconsistent findings on peripheral BDNF levels.

The incomplete handling of confounding factors represents another important limitation of the evidence. Antipsychotic use, a known modulator of peripheral BDNF levels, was acknowledged in five of the eight studies [25,26,28,29,35], whereas BMI was reported only by two [28,29,37]. Antidepressant exposure, despite its well-established influence on peripheral BDNF, was acknowledged and systematically addressed only in Yee et al. [29]. All but two studies [35,36] reported smoking status, but none considered other relevant behavioral factors such as physical exercise. This incomplete and heterogeneous handling of confounders likely contributed to the variability observed across studies and reduces the interpretability of their findings.

### 4.8. Limitations of This Review

This review has several limitations. Despite the use of multiple databases, the possibility of missing relevant studies and the influence of publication bias cannot be excluded. Only eight studies met the inclusion criteria, and their heterogeneity in design, populations, and outcome reporting limits the generalizability of the findings. Because of this small and diverse evidence base, a meta-analysis was not feasible, and the synthesis of results was necessarily narrative.

### 4.9. Implications for Practice and Future Research

Findings across the eight studies included in this review were mostly inconsistent, and no discernible pattern in peripheral BDNF levels among ultra-high-risk groups could be identified when compared with healthy controls or individuals with established psychotic disorders. These findings come from a relatively small number of methodologically heterogeneous studies. Taken together, they do not support any clinical application of peripheral BDNF levels in the early identification or management of psychosis.

Our recommendations for future research are informed by the limitations observed in the studies included in this review. Future studies should first prioritize larger, adequately powered cohorts to improve the reliability of cross-sectional comparisons and the detection of meaningful differences in peripheral BDNF levels between UHR populations and relevant comparison groups. For studies with prognostic aims, follow-up of adequate duration is essential to capture clinically meaningful transitions and evaluate the potential of BDNF as a longitudinal marker of disease progression. Methodological standardization should be encouraged, with protocols ensuring a consistent choice of biological matrix (serum or plasma), standardized sample collection methods, and validated assay techniques with uniform reporting. A more systematic approach to managing confounding factors is needed, as attention to them appears uneven. Variables such BMI, smoking, and antipsychotic use are commonly acknowledged. In contrast, antidepressant exposure and physical activity are less consistently accounted for, which can lead to biased or misleading findings on peripheral BDNF levels. The inconsistent findings observed to date may reflect the biological complexity of the neurotrophin pathway. Consequently, relying solely on peripheral BDNF levels may be insufficient; incorporating participant genotyping and additional markers (e.g., proBDNF, BDNF mRNA) could provide a more comprehensive understanding of early neurobiological changes associated with psychotic illness [32,47].

## Figures and Tables

**Table 1:** Overview of studies examining peripheral BDNF levels in individuals at high risk for psychosis (UHR), compared to healthy controls (HCs), patients with first-episode psychosis (FEP), and individuals with chronic schizophrenia (CS). DS-FEP: deficit schizophrenia—first episode psychosis, NDS-FEP: non deficit schizophrenia—first episode psychosis, PS: first diagnosis of any psychotic disorder except for substance-induced psychotic disorder and psychotic disorder due to a medical condition, established within the last five years. ^a^ Approximated values extracted from graphics. The authors did not provide exact numbers. * Statistically meaningful.

**Article**		**Heitz et al. (2018)**			**Loch et al. (2023)**				**Sanada et al. (2018)**				**Yee et al. (2018)**			
**Group**		UHR	FEP	CS	HC		UHR		HC		UHR		HC		UHR	
**Sample size (n)**		16	6	11	55		81		30		13		106		105	
**Mean BDNF (ng/mL)**	Serum	19.11 (±4.67)	24.48 (±2.40)	28.08 (±3.99)	_		_		_		_		3.3 (±1.5)		3.7 (±1.4)	
	Plasma	0.30 (±0.29)	0.54 (±0.54)	1.31 (±1.06)	0.48 (±0.37)		0.53 (±0.36)		12.92 (±10.40)		3.86 (±1.43)		_		_	
***p*-value**		Serum 0.015 * Plasma 0.09			0.596				<0.001 *				0.018 *			
**UHR selection criteria**		BSIP			SIPS				CAARMS				CAARMS			
**Average age (years)**		24.6	29.4	38.4	25.1		24.8		24		22.2		22		21.8	
**BMI (kg/m^2^)**		Unstated			Unstated				Unstated				22.3		22.5	
**Use of antipsychotics n° (%)**		1 (6.3)	4 (66.7)	11 (100)	Unstated				0 (0)		8 (61.5)		0 (0)		0 (0)	
**Use of antidepressants n° (%)**		Unstated			Unstated				Unstated				0 (0)		46 (43.8)	
**Use of nicotine or tobacco n° (%)**		11 (68.8)	4 (66.7)	11 (100)	18 (32.7)		26 (32.1)		5 (16.7)		8 (61.5)		19 (18.1)		31 (29.5)	
**Follow-up**		No			30 months				6 months to UHR participants				24 months			
**Article**		**Kelsven et al. (2020)**			**Counotte et al. (2019)**				**He et al. (2019)**				**Cecerska-Heryć et al. (2024)**			
**Group**		HC	UHR	FEP	HC	UHR		PS	HC	UHR		FEP	HC	UHR	FEP	CS
**Sample size (n)**		7	11	50	39	11		38	29	30		30	34	13	31	72
**Mean BDNF (ng/mL)**	Serum	_	_	_	18 ^a^	20 ^a^		19 ^a^	_	_		_	35 ^a^	21 ^a^	21 ^a^	19 ^a^
	Plasma	3.88 (±1.17)	18.08 (±8.67)	5.02 (±6.17)	_	_		_	11.56 (±1.06)	11.43 (±1.27)		12.59 (±1.25)	_	_	_	_
***p*-value**		<0.001 *			0.279				0.001 *				<0.01 * for HC and DS-FEP <0.05 * for HC and NDS			
**UHR selection criteria**		SIPS			CAARMS				SIPS				SIPS			
**Average age (years)**		20.4	18.5	24.1	24	24		25.5	20.4	19.8		19.6	36	23	29	39
**BMI (kg/m^2^)**		Unstated			22.8	23.1		23	Unstated				25.6	22.7	24.3	28.1
**Use of antipsychotics n° (%)**		0 (0)	0 (0)	10 (20)	0 (0)	0 (0)		0 (0)	Unstated				0 (0)	10 (76.9)	28 (90.3)	68 (94.4)
**Use of antidepressants n° (%)**		Unstated			Unstated				Unstated				Unstated			
**Use of nicotine or tobacco** **n° (%)**		Unstated			6 (15.4)	9 (81.8)		16 (42.1)	Unstated				0 (0)	0 (0)	4 (12.9)	23 (31.9)
**Follow-up**		No			No				No				No			

## Data Availability

Data is available in public databases used for this systematic review.

## Abbreviations

The following abbreviations are used in this manuscript:

BDNF	Brain-Derived Neurotrophic Factor
UHR	Ultra-high risk
HC	Healthy controls
FEP	First-episode psychosis
CS	Chronic schizophrenia
ARMS	At-risk mental state
APS	Attenuated positive symptoms
BLIPS	Brief limited intermittent psychotic symptoms
BMI	Body mass index
JBI	Joanna Briggs Institute
PRISMA	Preferred Reporting Items for Systematic Reviews and Meta-Analyses
CAARMS	Comprehensive Assessment of At-Risk Mental States
SIPS	Structured Interview for Prodromal Syndromes
LYRIKS	Longitudinal Youth-At-Risk Study
